# Role of blood cell-associated angiotensin II type 1 receptors in the cerebral microvascular response to ischemic stroke during angiotensin-induced hypertension

**DOI:** 10.1186/2040-7378-3-15

**Published:** 2011-11-16

**Authors:** Mutsumi Nagai, Satoshi Terao, Shantel A Vital, Stephen F Rodrigues, Gokhan Yilmaz, D Neil Granger

**Affiliations:** 1 Department of Molecular and Cellular Physiology, Louisiana State University Health Sciences Center, 1501 Kings Highway, Shreveport, LA 71130, USA

**Keywords:** angiotensin II type 1 receptor, blood brain barrier, bone marrow chimera, cerebral ischemia, inflammation

## Abstract

**Background:**

Angiotensin II type 1 receptor (AT1R) blockers lower the incidence of ischemic stroke in hypertensive patients and attenuate brain inflammation and injury in animal models. Although AT1R on both blood cells (BC) and vascular endothelial cells (EC) can be activated by angiotensin II (Ang II) to elicit inflammation, little is known about the relative contributions of AT1R expressed on BC and EC to the brain injury responses to ischemia and reperfusion (I/R) in the setting of angiotensin-induced hypertension.

**Methods:**

The contributions of BC- and EC-associated AT1R to I/R-induced brain inflammation and injury were evaluated using wild type (WT), AT1aR^-/-^, and bone marrow chimera mice with either a BC+/EC+ (WT→WT) or BC-/EC+ (AT1aR^-/-^→WT) distribution of AT1aR. The adhesion of leukocytes and platelets in venules, blood brain barrier (BBB) permeability and infarct volume were monitored in postischemic brain of normotensive and Ang II-induced hypertensive mice.

**Results:**

The inflammatory (blood cell adhesion) and injury (BBB permeability, infarct volume) responses were greatly exaggerated in the presence of Ang II-induced hypertension. The Ang II-enhanced responses were significantly blunted in AT1aR^-/- ^mice. A similar level of protection was noted in AT1aR^-/- ^→WT mice for BBB permeability and infarct volume, while less or no protection was evident for leukocyte and platelet adhesion, respectively.

**Conclusions:**

BC- and EC-associated AT1aR are both involved in the brain injury responses to ischemic stroke during Ang II-hypertension, with EC AT1aR contributing more to the blood cell recruitment response and BC AT1aR exerting a significant influence on the BBB disruption and tissue necrosis elicited by I/R.

## Background

The renin-angiotensin system (RAS) has been implicated in the pathogenesis of different cardiovascular (CV) diseases, including stroke. Angiotensin II (Ang II), the principal effector molecule of the RAS, exerts some deleterious effects on the vasculature of different organ systems via its pressor properties and the resultant elevation of blood pressure. An additional component of the vascular dysfunction and tissue injury response mediated by Ang II has been attributed to the ability of the peptide to induce a pro-inflammatory, pro-thrombogenic, and pro-oxidative phenotype in both large and microscopic blood vessels [1-3]. Both the vasomotor and inflammatory effects of Ang II involve the activation of angiotensin II type 1 receptors (AT1R) that are expressed on leukocytes and platelets, endothelial cells and vascular smooth muscle. Engagement of Ang II to AT1R results in the activation of these cells, and the consequent induction of oxidative stress and production and/or release of different inflammatory proteins, such as cytokines and adhesion glycoproteins. While endothelial cell-associated AT1R has received much attention as an integral component of the pathophysiology of different CV diseases, recent studies have revealed that AT1R expressed on bone marrow (BM)-derived cells can also make a significant contribution to disease pathogenesis. For example, it has been reported that apolipoprotein E-deficient (ApoE^-/-^) BM chimeric mice with AT1R^-/- ^blood cells and AT1R^+/+ ^vessel wall components exhibit blunted Ang II-enhanced atherosclerosis, comparable to that noted in AT1R^-/-^/ApoE^-/- ^double knockout mice, suggesting that AT1R on BM derived cells is critical for the progression of atherosclerosis [4]. Similarly, blood cell-associated AT1R has been implicated in the vasomotor dysfunction that accompanies Ang II-induced hypertension [5] and the enhanced inflammatory cell adhesion observed in venules of hypercholesterolemic mice [6] while recent report showed the protective effect of BM-derived cells on Ang II-induced hypertension [7].

Angiotensin II has been implicated in the pathogenesis of ischemic and hemorrhagic stroke. Animal studies have revealed that AT1R blockers blunt the brain inflammation and tissue injury response to ischemia and reperfusion (I/R) [8]. Reduced ischemic lesion areas after brain I/R have also been detected in mice that are genetically deficient in either AT1R [9,10] or angiotensinogen [11], while an exaggerated brain injury response (mediated through AT1R) has been noted in renin/angiotensinogen transgenic mice [12,13]. Acute [14] or chronic [15,16] administration of Ang II in WT mice has been shown to induce the adhesion of leukocytes and platelets, and compromise blood brain barrier (BBB) function in the cerebral microvasculature. These responses to exogenous Ang II were dependent on AT1R activation and the production of reactive oxygen species, but independent of the elevated blood pressure. In the presence of exogenously administered Ang II, I/R elicit more intense brain inflammatory and tissue injury responses that are prevented by pharmacological AT1R blockade [14]. While the existing literature strongly implicates AT1R in the brain injury resulting from ischemic stroke in both normotensive and Ang II-hypertensive animals, the cell populations that contribute to the AT1R-dependent cerebral microvascular dysfunction and brain injury response to I/R remain undefined.

Gene cloning studies of AT1R have revealed that mice and rats have two subtypes of AT1R (AT1aR and AT1bR) whereas human have only one receptor type [17]. The functions of the two receptors found in rodents are largely indistinguishable, with AT1aR making a more substantial contribution to blood pressure regulation [18]. Consequently, AT1aR knockout mice have been widely employed to assess the physiologic and pathophysiologic roles of the angiotensin II type 1 receptors [3,6].

Hence, the overall objective of this study was to determine the relative contributions of AT1R expressed on blood cells vs endothelial cells to the brain injury responses elicited by I/R in both normotensive and Ang II-hypertensive mice. This objective was achieved by comparing the injury and inflammatory responses of the cerebral microvasculature to I/R in WT and AT1aR^-/- ^mice to the responses elicited in AT1aR^-/- ^BM chimeras that were absent AT1aR on BM-derived cells while expressing AT1aR on the vascular wall. Our findings are consistent with injury response-specific contributions of both blood cell- and vascular wall associated AT1aR to ischemic stroke in both normotensive and Ang II-hypertensive mice.

## Methods

### Animals

Male C57Bl/6J WT mice (n = 120), AT1aR^-/- ^mice (B6.129P2-*Agtr^1tm1Unc^*/J) (Jackson Laboratories Bar Harbor) (n = 35), and BM chimeric mice (n = 81) were studied. All experimental procedures involving the use of animals were reviewed and approved by the Institutional Animal Care and Use Committee of LSU Health Sciences Center and performed according to the Guide for the Care and Use of Laboratory Animals outlined by the National Institutes of Health.

### BM Chimeras

WT to WT (WT→WT, n = 41) and AT1aR^-/- ^to WT (AT1aR^-/-^→WT, n = 40) BM chimeras were produced, as previously described [15], by transplanting BM from either WT or AT1aR^-/- ^into WT mice. In brief, BM cells, collected from the femurs and tibias of donor mice were injected (2 × 10^6 ^BM cells) via the femoral vein into recipient mice (congenic WT with same phenotype as C57BL/6J mice; B6.SJLPtprc^a^Pep3^b^/BoyJ), following total-body irradiation sufficient to eliminate the recipient's blood cells. The BM chimeras were housed in autoclaved cages, with 0.2% neomycin added to drinking water for the first two weeks. After six to eight weeks, reconstitution of BM cells was verified using flow cytometry by testing for the % blood leukocytes positive for CD45.1 (recipient isoform of CD45) vs CD45.2 (donor isoform of CD45). We used BM chimeras in which > 90% of recipient marrow was replaced by donor BM.

### Angiotensin II infusion

Mice received a 14-day infusion of either saline or Ang II (Bachem Americas, Inc.) using micro-osmotic pumps (Durect Corporation), which were implanted subcutaneously in the intrascapular region under isofluorane anesthesia. Sterile procedures were used and a topical antibiotic Neosporin (Johnson & Johnson) was applied to prevent postoperative infection at the site of implantation. The pumps in the control group were loaded with only the saline vehicle, while the experimental groups received Ang II loaded pumps that delivered the peptide at a rate of 2000 ng/kg/min. In some experiments, a range of Ang II doses (400 - 2000 ng/kg/min) were used to determine the dose-dependent responses of leukocyte and platelet adhesion, and blood pressure to Ang II in the absence of I/R.

### Blood pressure

Measurements of blood pressure in conscious mice were obtained using a tapered femoral artery catheter implanted (under isofluorane anesthesia) three hours prior to data collection via a pressure transducer coupled to a computer system, as previously described [15]. Mean arterial pressure was recorded over the 1-hour period preceding the experiment.

### Middle cerebral artery occlusion and reperfusion

Transient (45 minutes) focal cerebral ischemia was induced in ketamine (100 mg/kg, i.p., Lloyd Laboratories) and xylazine (10 mg/kg, i.p., Hospira, Inc.) anesthetized mice by occluding the right middle cerebral artery with 7-0 silicone-coated nylon monofilament (Doccol Corp.). After the 45-minute occlusion period, the nylon fiber was gently removed and the common carotid artery was reopened [19,20]. In sham mice, the arteries were visualized but not disturbed. Ischemia and reperfusion were verified using a laser Doppler flowmeter (ML191 Blood Flow Meter, ADInstruments) to monitor cerebral blood flow. We have demonstrated that middle cerebral artery occlusion is associated with a 90% reduction in brain perfusion [21]. Core temperature was kept at 36 - 37°C.

### Intravital videomicroscopy

Four hours following reperfusion after middle cerebral artery occlusion, mice were anesthetized with ketamine (100 mg/kg, i.p.) and xylazine (10 mg/kg, i.p.) then prepared for intravital microscopic observation and evaluation of the cerebral microvasculature, as previously described [22,23]. Briefly, cerebral venules (100 μm length, 25-50 μm diameter) were viewed through a cranial window using an upright fluorescent microscope using a 20× water immersion lens. Color images were captured with a 3 charge coupled device color video camera. Ex vivo carboxyfluorescein diacetate succinimidyl ester (CFDSE) (Molecular Probes, Invitrogen) labeled platelets (100 × 10^6 ^cells) derived from a matching donor mouse were administered for visualization and quantification of (green) adherent platelets. Rhodamine 6G (Sigma Chemical) was administered for detection of leukocytes (red). Adherent leukocytes and platelets were defined as cells bound to the venular wall for ≥ 30 and 2 seconds, respectively [6,15]. Cell adhesion data are expressed as number of cells per millimeter squared of venular surface, calculated from venular diameter and length, assuming cylindrical geometry.

### Infarct Volume

After 24 hours of reperfusion, mice were decapitated under deep anesthesia with ketamine (200 mg/kg, i.p.) and xylazine (20 mg/kg, i.p.) then 1-mm-thick coronal sections of the brain were immersed in 0.05% 2,3,5-triphenyltetrazolium chloride (TTC, Sigma-Aldrich) solution for 30 minutes. The total areas of each brain section and the infarct region were quantified with the software program, NIH image. Infarct volume was corrected for edema as previously described [23].

### BBB Dysfunction

Changes in the barrier properties of the cerebral microvasculature were monitored using the Evans blue (EB) extravasation method, as previously described [19,20]. A 2% solution of EB (Sigma-Aldrich) was administered (4 ml/kg, i.v.) immediately following I/R or after sham operation. Twenty-four hours later, mice were anesthetized with ketamine (100 mg/kg, i.p.) and xylazine (10 mg/kg, i.p.) then plasma sample was collected and brain tissue (right hemisphere with brain stem and dura mater removed) sample was collected after transcardial perfusion with PBS (100 mm Hg, 5 minutes). EB concentrations were determined using a fluorescence spectrophotometer (FLUOstar Optima; BMG LABTECH, Inc.). BBB permeability was estimated by dividing tissue EB concentration (ng/g brain weight) by the plasma concentration (ng/ml).

### Statistical Analysis

All of the values are reported as mean ± SE. One-way analysis of variance with Fisher's post-hoc test was used to determine statistical differences between groups. Statistical significance was set at *P *< 0.05.

## Results

### Blood pressure responses to chronic Ang II infusion

**Figure 1:**
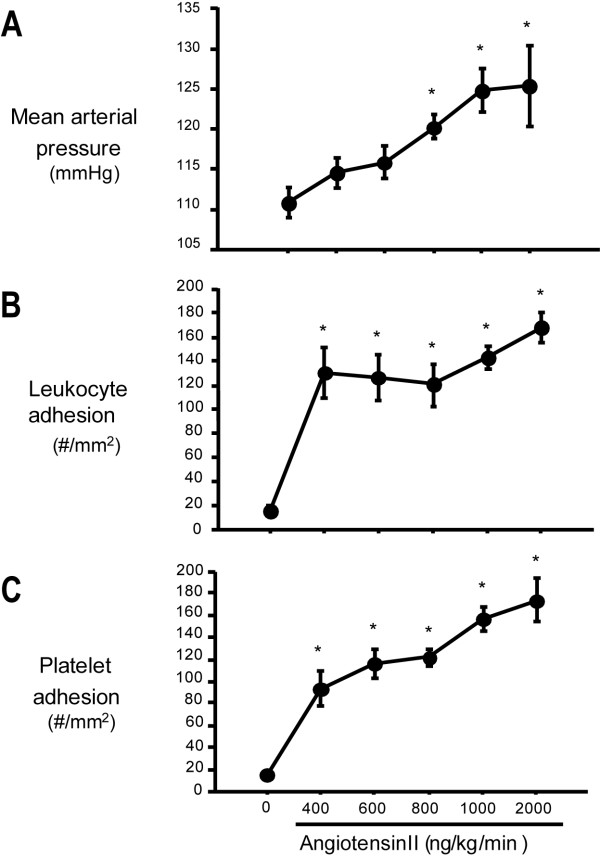
**Chronic Ang II infusion elicits dose-dependent increases in blood pressure and the adhesion of leukocytes and platelets in cerebral venules**. WT mice received a 14-day infusion of Ang II subcutaneously in pumps loaded to deliver either 0, 400, 600, 800, 1000, or 2000 ng/Kg/min. (**Panel A**): Mean arterial blood pressure was measured in conscious mice with an indwelling femoral arterial catheter. (**Panels B & C**): The adhesion of leukocytes (**B**) and platelets (**C**) in cerebral venules was monitored via a cranial window by fluorescence intravital videomicroscopy. Leukocytes were labeled in vivo with Rhodamin 6G while platelets were labeled with CFDSE ex vivo. Data are shown as mean ± SEM for 6-13 mice per Ang II dose. * denotes p < 0.05 relative to saline infusion.

A two-week infusion of Ang II produced a dose-dependent increase mean arterial pressure in WT mice (Figure 1A), with statistically significant increases noted at Ang II infusion rates between 800 and 2000 ng/kg/min, compared to saline infusion (controls). The increased blood pressure induced by 2000 ng/kg/min Ang II in WT mice, was not observed in either AT1aR^-/- ^or AT1aR^-/-^→WT mice (Table 1).

**Table 1:** Chronic angiotensin II infusion (2000 ng/kg/min) induces hypertension via blood cell associated AT1aR.

*Experimental group*	*Mean arterial pressure* (mmHg)
**Wild type mice (WT) - saline loaded (- saline)** (n = 9)	110.8 ± 1.8

**WT - angiotensin II loaded (- Ang II)** (n = 10)	125.4 ± 4.9*

**AT1aR knockout mice (AT1aR^-/-^) - Ang II** (n = 6)	103.4 ± 4.0^†^

**WT bone marrow chimera mice reconstructed with WT bone marrow (WT→WT) - saline** (n = 7)	96.6 ± 2.0

**WT→WT - Ang II** (n = 8)	115.1 ± 6.1^‡^

**AT1aR^-/-^→WT - Ang II** (n = 6)	108.9 ± 3.2^†^

Data are presented as mean ± SE. * denotes P < 0.05 vs WT - saline, † denotes p < 0.05 relative to WT - Ang II, ‡ denotes P < 0.05 vs WT→WT - saline

### Chronic Ang II infusion promotes leukocyte-endothelial cell adhesion in cerebral venules

In WT mice not subjected to cerebral I/R, chronic Ang II infusion resulted in a large and significant increase in the number of adherent leukocytes, the magnitude of which did not differ between Ang II infusion rates of 400 and 2000 ng/kg/min (Figure 1B).

### Roles of blood cell vs vascular wall AT1aR in cerebral I/R-induced leukocyte adhesion in normotensive and Ang II-hypertensive mice

**Figure 2:**
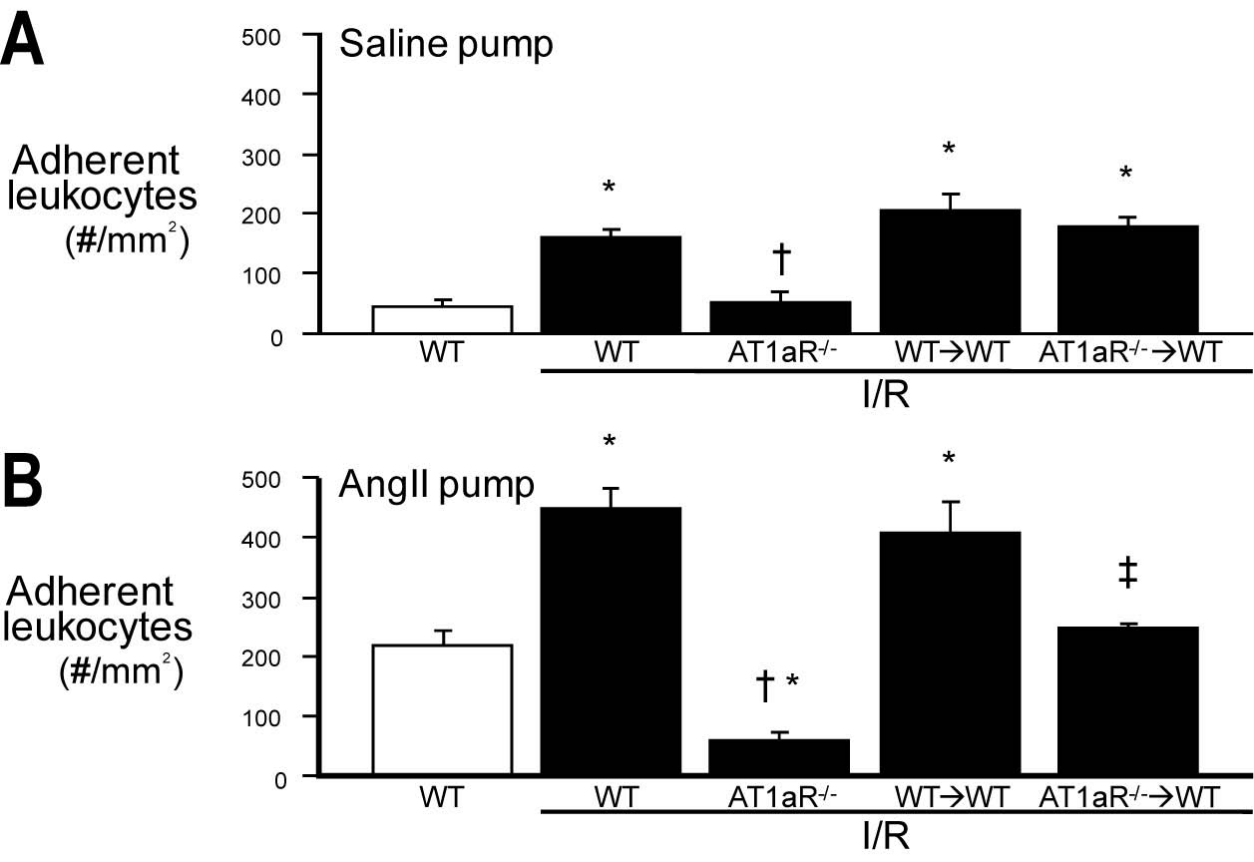
**Cerebral I/R induces an AT1aR-dependent leukocyte recruitment response that is mediated by vascular wall-associated AT1aR in normotensive mice, while blood cell-associated AT1aR mediates the response in Ang II hypertensive mice**. Focal brain ischemia (45 min) and reperfusion (4 hrs) were induced by occluding the middle cerebral artery using the intraluminal filament method. Leukocyte-endothelial cell adhesion in cerebral venule was monitored through a cranial window by fluorescence intravital videomicroscopy. Angiotensin (2000 ng/kg/min)(**Panel B**) or an equal volume of saline (**Panel A**) was infused over a 2 wk period in WT, AT1aR^-/-^, WT→WT BM chimeric, and AT1aR^-/-^→WT BM chimeric mice. Leukocytes were labeled in vivo with Rhodamin 6G for fluorescence. Data are shown as mean ± SE with 5-7 mice per group. * denotes p < 0.05 relative to corresponding non-ischemic WT mice. † denotes p < 0.05 relative to corresponding WT mice + I/R. ‡ denotes p < 0.05 relative to corresponding WT→WT BM chimeras.

In WT mice with a saline-loaded Alzet pump (Figure 2A), focal cerebral I/R elicited a significant increase in leukocyte adhesion, which was not observed in AT1aR^-/- ^mice. The leukocyte adhesion response in AT1aR^-/-^→WT mice did not differ from the WT→WT response. The findings are consistent with a role for vessel wall-, but not blood cell-, associated AT1aR in mediating the leukocyte recruitment elicited by cerebral I/R.

In WT mice with an implanted Ang II-loaded pump (Figure 2B), the numbers of adherent leukocytes detected in cerebral venules were much higher than observed in their saline pump counterparts, both in the absence or presence of I/R. The significant increase in leukocyte adhesion elicited by I/R during chronic Ang II infusion was not observed in AT1aR^-/- ^mice. Unlike the saline pump mice, AT1aR^-/-^→WT mice exhibited a significant but partial reduction in leukocyte adhesion compared to their WT→WT counterparts. These findings indicate that both vessel wall- and blood cell-associated AT1aR contribute to the leukocyte recruitment response to I/R during chronic Ang II infusion.

### Platelet adhesion responses to chronic Ang II infusion: Influence of I/R and AT1aR

**Figure 3:**
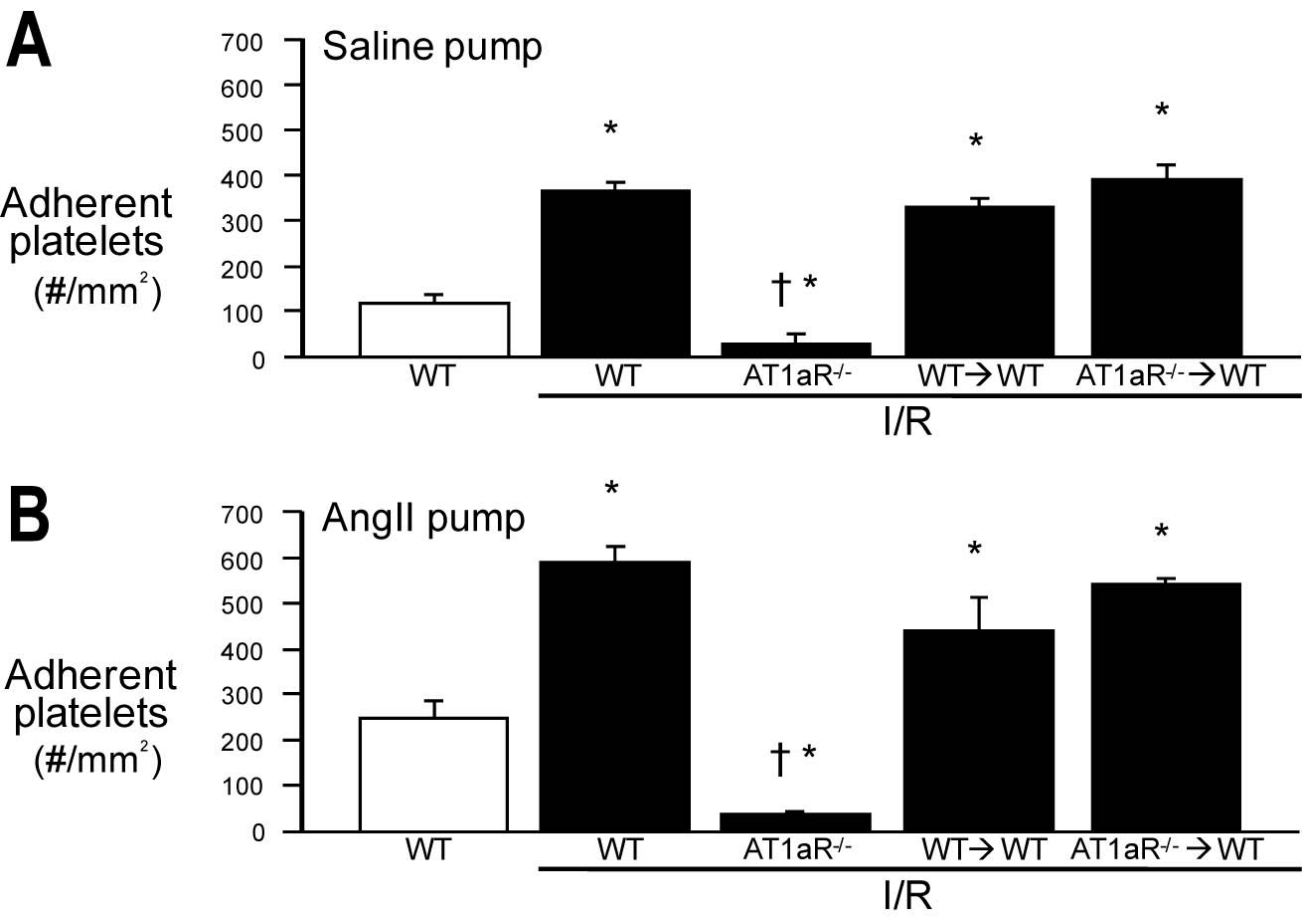
**Cerebral I/R elicits AT1aR dependent platelet recruitment, with vascular wall-associated AT1aR mediating the response in both normotensive and Ang II-induced hypertensive mice**. Focal brain ischemia (45 min) and reperfusion (4 hrs) were induced by occluding the middle cerebral artery using the intraluminal filament method. Platelet adhesion in cerebral venule was monitored through a cranial window by fluorescence intravital videomicroscopy. Angiotensin (2000 ng/kg/min)(**Panel B**) or an equal volume of saline (**Panel A**) was infused over a 2 wk period in WT, AT1aR^-/-^, WT→WT BM chimeric, and AT1aR^-/-^→WT BM chimeric mice. Platelets were labeled ex vivo with CFDSE. Data are shown as mean ± SE with 5-7 mice per group. * denotes p < 0.05 relative to corresponding non-ischemic WT mice. † denotes p < 0.05 relative to corresponding WT mice + I/R. n = 5-7 mice per group.

In WT mice not subjected to cerebral I/R, chronic Ang II infusion resulted in a dose-dependent increase in the number of adherent platelets (Figure 1C). In a manner similar to leukocyte adhesion, I/R elicited significant increases in platelet adhesion in WT mice receiving either a saline or Ang II infusion (Figure 3A &3B). Unlike the leukocyte adhesion responses to I/R, a significant attenuation in platelet adhesion was noted after I/R only in AT1aR^-/- ^mice, with no differences noted between WT→WT and AT1aR^-/-^→WT mice during either saline or Ang II infusion. These findings suggest that vessel wall-, but not blood cell-, associated AT1aR contributes to the I/R-induced platelet recruitment response in the presence or absence of chronically elevated Ang II levels.

### Alterations in BBB function induced by I/R during chronic Ang II infusion

**Figure 4:**
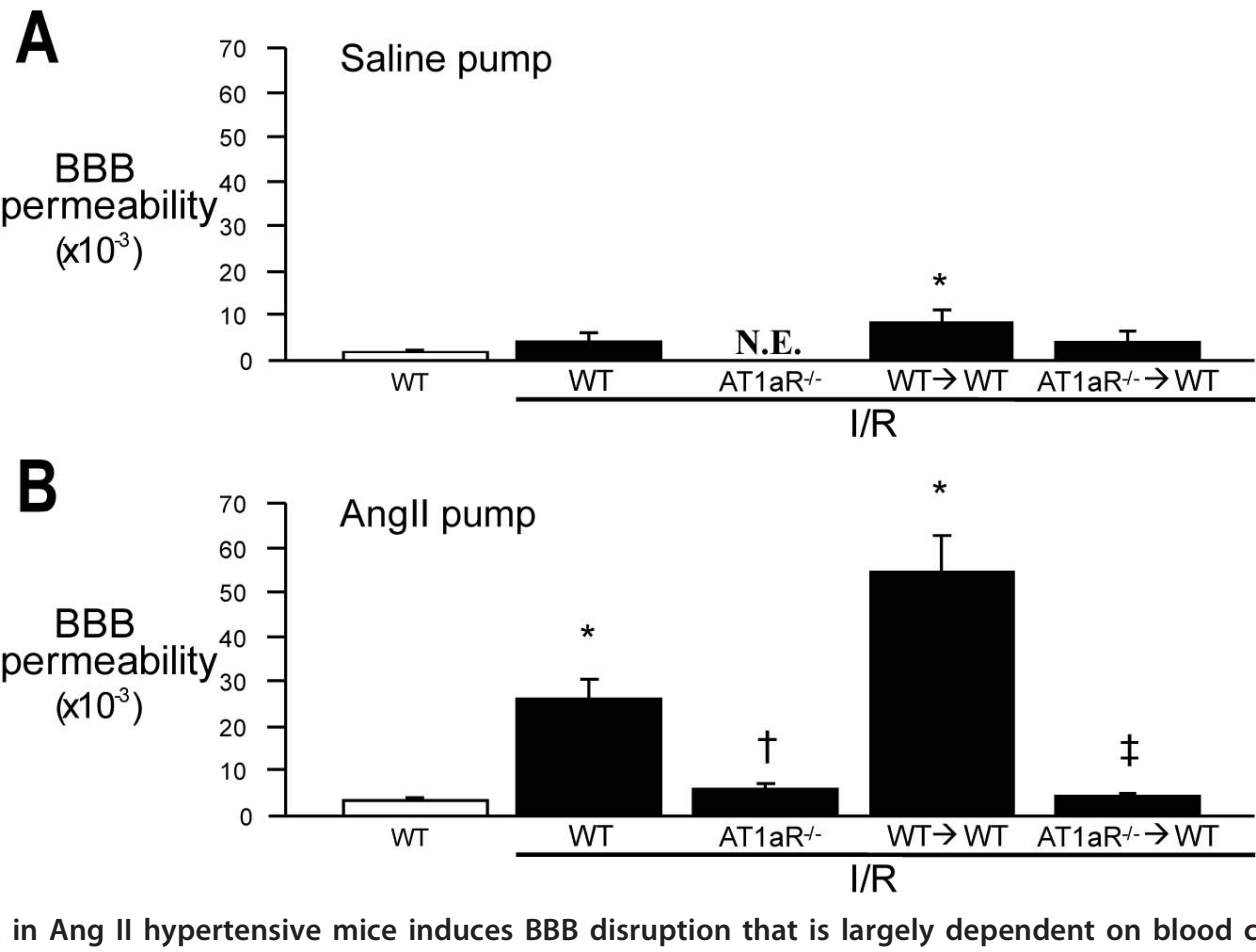
**Cerebral I/R in Ang II hypertensive mice induces BBB disruption that is largely dependent on blood cell-associated AT1aR**. Focal brain ischemia (45 min) and reperfusion (24 hrs) were induced by occluding the middle cerebral artery using the intraluminal filament method. Evan's blue (EB) dye was administered at the time of reperfusion or sham operation. Twenty-four hours later, brain tissue and plasma samples were collected for EB measurement. BBB permeability was estimated by dividing tissue EB concentration by plasma concentration in saline (**Panel A**) or Ang II (2000 ng/kg/min, 2 wks)(**Panel B**) infused mice. The data are shown as mean ± SE with 5-7 mice per experimental group. * denotes p < 0.05 relative to non-ischemic WT mice. † denotes p < 0.05 relative to WT mice + I/R. ‡ denotes p < 0.05 relative to WT→WT BM chimeras. N.E.: no examination.

Figure 4A summarizes the changes in BBB permeability following I/R in mice implanted with a saline pump. The data shows a tendency for increased BBB permeability following I/R, which was more pronounced (and statistically different) in the WT > WT chimeras compared to WT controls. In mice receiving a chronic infusion of Ang II (Figure 4B), a large and significant increase in BBB permeability was noted after I/R. The I/R-induced EB leakage was entirely prevented in both AT1aR^-/- ^and AT1aR^-/-^→WT mice, suggesting that blood cell-associated AT1aR accounts for the impaired endothelial function elicited by I/R in the presence of chronically elevated Ang II levels. The trend for WT > WT chimeras to exhibit a more robust response to I/R than WT mice was more evident (and statistically significant) in the presence of AngII infusion. Both the saline and AngII infusion responses therefore suggest that the bone marrow transplantation procedure may predispose the cerebral microvasculature to a more exaggerated response to an inflammatory stimulus, like I/R. This possibility is consistent with the findings of published report [24].

### Contributions of Ang II and AT1aR to tissue infarction elicited by cerebral I/R

**Figure 5:**
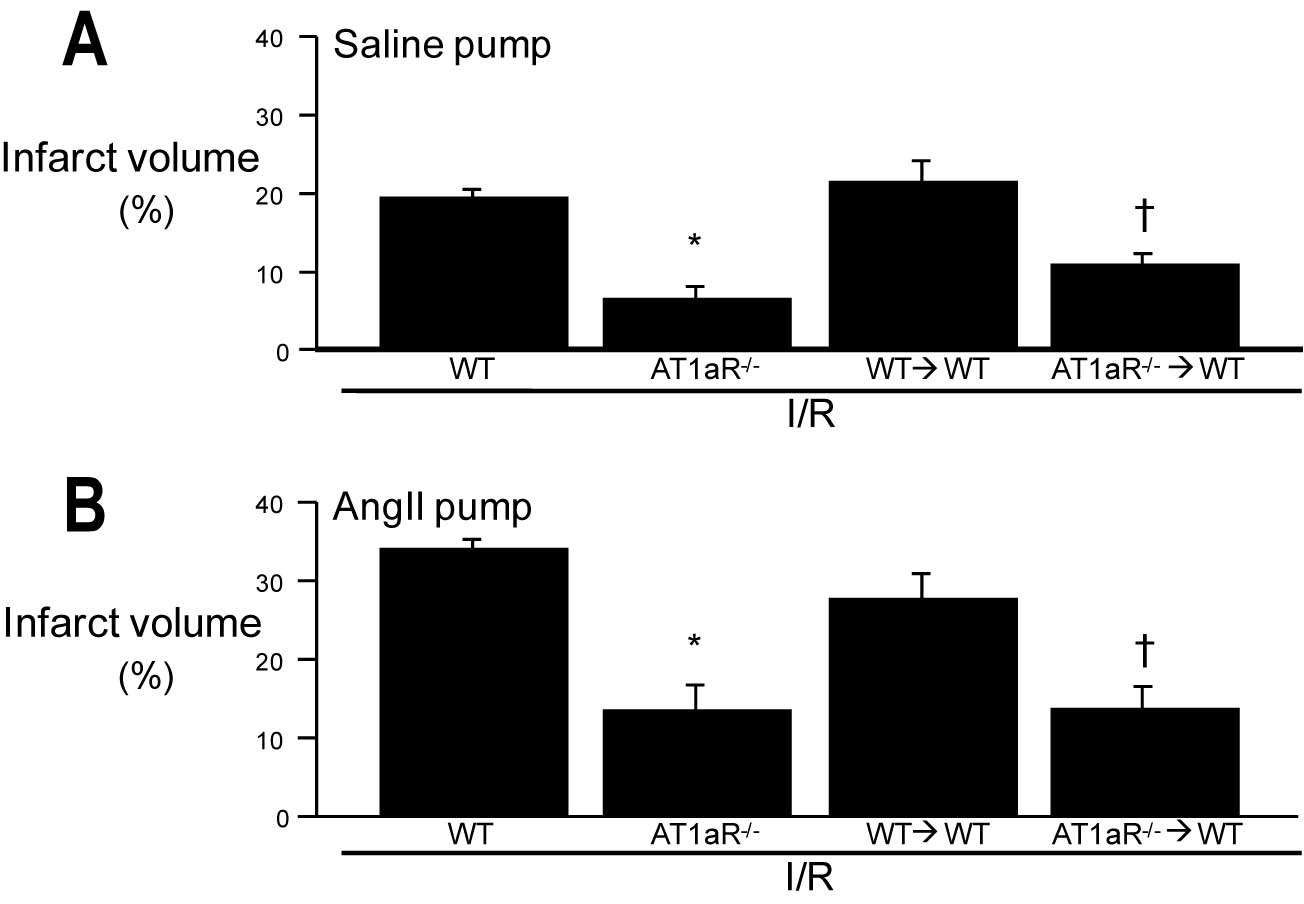
**The brain infarction induced by cerebral I/R in both normotensive and Ang II hypertensive mice is largely dependent on blood cell associated-AT1aR**. Twenty four hours following focal cerebral ischemia (45 min), brain tissue slices were stained with TTC and cerebral infarct volume was determined (using imaging software) from the regions not stained with the vital dye. This procedure was applied to brain sections obtained from Ang II (2000 ng/kg/min, 2 wks) or saline infused WT, AT1aR^-/-^, WT→WT BM chimeric and AT1aR^-/-^→WT BM chimeric mice. Data are shown as mean ± SE with 5-6 mice per group. * denotes p < 0.05 relative to corresponding WT mice + I/R. † denotes p < 0.05 relative to corresponding WT→WT BM chimeras.

Focal cerebral I/R produced significant brain infarcts in both saline- and Ang II-infused mice, with the Ang II group exhibiting a 77% larger infarct (p < 0.05) than the saline group (Figure 5A &5B). Highly significant and nearly identical reductions in infarct volume were noted in AT1aR^-/- ^and AT1aR^-/-^→WT mice subjected to I/R in both saline- and Ang II-infused mice. These findings suggest that blood cell-associated AT1aR are largely responsible for the Ang II mediated tissue injury observed following cerebral I/R in the absence or presence of chronically elevated Ang II levels.

## Discussion

There are several published reports that implicate the RAS in the pathogenesis of ischemic stroke. Clinical and basic studies have revealed the ability of AT1R blockers to reduce the incidence and protect tissue injury from stroke independent of the blood pressure-lowering effect [25-29], while animal studies demonstrate attenuated inflammatory and injury responses in post-ischemic brain following pharmacologic blockade or genetic deletion of AT1R [8]. These findings have often been interpreted to reflect the pro-inflammatory response that results from the activation of AT1R on vascular endothelial cells [14,30]. However, recent studies of Ang II-induced hypertension [5,7] and Ang II-accelerated atherogenesis [4] have revealed a role for blood cell-associated AT1R in mediating the inflammation and vascular dysfunction/injury in these models of human disease. The results of the present study provide new insights regarding the relative contributions of blood cell- and vessel wall-associated AT1aR to the brain injury responses elicited by focal cerebral I/R in both normotensive and Ang II-hypertensive mice.

Our finding that chronically elevated Ang II levels results in an exaggerated brain injury response to I/R and that genetic deficiency of AT1aR^-/- ^protects both normotensive and Ang II-hypertensive mice against the cerebral microvascular inflammation and tissue damage elicited by I/R are consistent with the existing literature [9,11-13]. However, our comparison of results from AT1aR^-/- ^mice to AT1aR^-/-^→WT BM chimeras reveal distinct roles of vessel wall- and blood cell-associated AT1aR as mediators of the blood cell recruitment and brain injury responses to I/R. In both normotensive and Ang II-hypertensive mice, vessel wall-associated AT1aR appears to be largely responsible for promoting the recruitment of leukocytes and platelets in cerebral microvessels. This observation is consistent with reports that describe endothelial cell responses to AT1R activation that tend to induce a pro-adhesive surface on endothelial cells, including a reduced expression of endothelial nitric oxide synthase [31], an increased production/release of reactive oxygen species [1,32] and cytokines [33], and an increased expression of endothelial cell adhesion molecules [34,35]. The dominant role of vessel wall AT1aR in mediating the I/R-induced leukocyte- and platelet-endothelial cell interactions may also reflect an influence of locally high levels of Ang II generated by brain parenchyma, which would exert less influence on AT1aR on BM-derived cells [27]. This could also explain why we detected a contribution of blood cell-associated AT1aR in I/R-induced recruitment of leukocytes (but not platelets) in mice receiving exogenous Ang II.

In contrast to the inflammatory cell recruitment elicited by cerebral I/R, the brain injury response, manifested as an increased BBB permeability and increased infarct size, appears to be mediated exclusively by AT1aR expressed on blood cells. This dependency of the I/R-induced injury response on blood cell-associated AT1aR was evident especially in Ang II-hypertensive mice. Recent *in vitro *[36] and *in vivo *[15] experiments suggest that exposure of the cerebral microvasculature (or monolayers of cultured cerebral microvascular endothelial cells) to Ang II (in the absence of I/R) results in an impairment of BBB function that is mediated by endothelial AT1R. The absence of a role for endothelial cell AT1aR in mediating the increased BBB permeability following I/R in Ang II-hypertensive mice suggests that either endothelial AT1aR are down-regulated or become unresponsive to Ang II, and/or that the direct effects of endothelial AT1aR activation are overwhelmed by the actions of chemical mediators released as a result of AT1aR activation on blood cells including resident macrophages as well as circulating cells [37]. The beneficial effects of blood cell AT1aR deficiency could also reflect the actions of Ang II on angiotensin II type-2 receptors, which have been shown to exert significant neuroprotective effects against stroke when stimulated on BM stromal cells [38].

While several previous studies have implicated the recruitment of leukocytes and platelets in the brain injury response to focal I/R [39,40], the results of the present study indicate that the blood cell recruitment and injury responses do not parallel one another when BM-derived cells are devoid of AT1aR (in BM chimeras). The exact mechanisms by which AT1aR expressed on BM derived cells mediate the tissue damage elicited by focal ischemic stroke remain unclear. However, these findings indicate that future work is needed to identify the specific blood cell population that mediates the deleterious effects of AT1aR activation in ischemic stroke and suggest that strategies that target blood cell-associated AT1aR may prove beneficial in prevention or treatment of ischemic stroke and other CV diseases.

## Conclusion

Our comparison of the injury and inflammatory responses of the cerebral microvasculature to I/R in wild type, AT1aR^-/-^, and AT1aR^-/-^→WT chimeras has revealed injury response-specific contributions of both blood cell- and vascular wall associated AT1aR. The results also indicate that AT1aRs expressed on BM-derived cells play a major role in mediating the brain inflammation and injury following ischemic stroke in both normotensive and AngII-hypertensive mice. Additional work is needed to define the mechanism(s) that underlie the brain injury response to AT1R activation on BM-derived cells.

## List of abbreviations used

**Ang II**: angiotensin II; **AT1R**: angiotensin II type 1 receptor; **ApoE^-/-^**: apolipoprotein E-deficient; **AT1aR^-/-^→WT**: AT1aR^-/- ^to WT; **BBB**: blood brain barrier; **BC**: blood cells; **BM**: bone marrow; **CFDSE**: carboxyfluorescein diacetate succinimidyl ester; **CV**: cardiovascular; **EC**: endothelial cells; **EB**: Evans blue; **I/R**: ischemia and reperfusion; **RAS**: renin-angiotensin system; **TTC**: 2,3,5-triphenyltetrazolium chloride; **WT**: wild type; **WT→WT**: WT to WT.

## Competing interests

The authors declare that they have no competing interests.

## Authors' contributions

MN performed animal experiments, collected and analyzed the data, and drafted the manuscript. ST also performed animal experiments, collected and analyzed data, and contributed to the manuscript. SAV produced bone marrow chimera mice, monitored blood pressure in conscious mice, and collected cell adhesion data. SR collected and analyzed data from the TTC and EB assays. GY collected and analyzed data from the EB assay. DNG conceived of the study, participated in its design and coordination, and helped draft the manuscript. All authors read and approved the final manuscript.
